# Touch of Medical Ethics at the Beginning of Medical School

**DOI:** 10.31729/jnma.8597

**Published:** 2024-05-31

**Authors:** Nova Ghimire, Dhirendra Yadav

**Affiliations:** 1 Patan Academy of Health Sciences, Lagankhel, Lalitpur, Nepal

**Keywords:** *curriculum*, *malpractice*, *medical ethics*, *medical school*, *medical students*

## Abstract

There comes a great responsibility to make patients feel better with the title of doctor. Often we meet certain moral and ethical dilemmas that cannot be solved by our medical textbook alone. It requires a certain assistance which can only be provided by medical ethics. Medical ethics is the guiding moral that guides Health Care Workers to provide a holistic approach while treating a patient.

Medical ethics is the soul that can not only prevent future dilemmas but can overall make a healthcare worker 'feel' and have empathy for the patient. From the story of Paul and the true life stories of patients we encountered during our visit to the Leprosy center, we got a sense of empathy.

Ethical practice and empathy are the pillars that can play a vital role in the rapidly increasing level of violence against the Health care professionals. So it is an emerging need to have medical ethics as a part of the curriculum among medical students.

## INTRODUCTION

Ethics is a branch of knowledge that differentiates between right and wrong. From the earliest centuries (aka Socrates) it has been a guiding light for people to follow what is right and provide justice.^1^ Medical ethics has been defined as principles that provide insight and guide medical personnel to have good judgment while practicing medicine.^2^

Medical ethics is based on four pillars that are Autonomy, Beneficence, non-maleficence, and Justice.^2^ These pillars are the foundation on which the decision of every medical profession rests upon.

## EXPERIENCES

Among the fortunate students, we had the opportunity to learn about medical ethics right from the start of medical school. Since the start of medical school, we have had several experiences that, in our opinion, have greatly aided in our ability to be in touch with our emotions. The field trip to Anandaban Hospital, which is part of The Leprosy Mission, is something we still reminisce about. The Leprosy Mission (TLM), an international non-governmental organization with headquarters in the UK, began operating in Nepal in 1957 when the Anandaban Hospital was established in Lele, Lalitpur.^3^ There we got to meet the people currently undergoing treatment for leprosy. We vividly remember how we were scared at first, having learnt previously that the disease could be transmitted, We too would have stayed away from them if not for our teacher who taught us that people undergoing treatment carry very low risk for transmission. Then as We got over our fear, We started communicating with them. We learnt that many of them were sent here by their family members because of a similar mindset that we had earlier i.e the fear of transmission. That made us see their pain in a different light. Then by slowly opening up they taught us that they are not a part of disease, they are humans with emotions and their emotions are also in dire need of being addressed along with their physical symptoms. By the end of the field trip we learned a valuable lesson that the beauty of human life and all its aspects is fragile and it will be our duty as future medical doctors to treat each individual as a person rather than a only case.

While reading the the autobiography of Paul Kalanithi ("When breath becomes air"), we came across a captivating story of a neurosurgeon battling with stage four of lung cancer. Even doctors who are granted the power to save lives are merely humans and might get sick, can suffer equally as others. It was depressing to read about Paul's struggle with the illness and the accompanying inner anguish and sorrow.^4^ We discovered that it is our responsibility as medical professionals to ensure the patient has a comfortable and painless death with dignity.. It made us realize that even in the last few hours it is our responsibility to listen to the patient and respect their choices even if it is against all our personal viewpoints. For example here Paul wanted to take his last breath as painlessly as possible (he chose comfort care) but we could have prolonged his life by resuscitating him but reading his story, we got to realize that this would have only prolonged his suffering. So from these classes on Medical ethics, we could see and relate to his point of view for a good death.

These are a few of the many experiences that have provided us with visions on how we could improve not only as a doctor but as a human being too. These classes evoked something in us during the early days of medical school that has left imprints on us till date.

From learning autobiography to presenting cases we got to observe in OPD we learnt the true value of empathy, humanity and true meaning behind the pillars of medical ethics.

## LEARNING

Medical ethics is not just a math equation with a definitive answer, there are the Grey zones. Grey zones i.e. the medical dilemmas is where there are conflicting choices and decisions for which these four pillars are of utmost importance.^5^ Sometimes the option that patient chooses may go beyond the principle of physician but it is something that the latter must respect. For example Euthanasia, the practice of intentionally ending life to eliminate pain and suffering, that is allowed in many countries including Switzerland might go against the main aim of doctors i.e. to treat and prevent death.^6^ But in this case, respecting patient wish i.e. autonomy should be above anything else. This is one of the many examples that only ethics can teach medical students.

It instructs us to attempt to reason through and weigh all of our options before reaching a decision. Like in the case of Paul, who after being given many options by his doctor, chose comfort care, and took his final peaceful breath. So medical ethics is the core that can help medical students understand that patients are not merely defined by their disease but also that their values that need to be addressed and respected.

## WAY FORWARD

Even though medical ethics are crucial to the clinical practice of medicine, many medical colleges still do not include it in their curriculum. It is becoming more and more necessary in the field of medicine to integrate ethics as a course in order to become better doctors and avoid future disasters, as Mahatma Gandhi once remarked, "Future depends on what we do in the present."

In their area of work, medical professionals routinely encounter moral and ethical quandaries. So adding medical ethics as a part of curriculum will provide new insight and offer a framework to assist them in reaching morally sound decisions for the patient in question.

Thus it can immensely help medical students to be shaped into kind doctors in the future who will know the true value of life and treat patients with empathy and use these knowledge, before making any difficult decision. Therefore, it is very beneficial to learn about medical ethics as early as possible since this will help us become better doctors, decision makers, and humans in general.
